# Supplementary material for: Asymmetric Synthesis of Quaternary Hydantoins via a Palladium-Catalyzed Aza-Heck Cyclization
Source: J Am Chem Soc. 2025 Nov 14;147(49):44692–8. doi: 10.1021/jacs.5c16022 (PMC12703750; doi:10.1021/jacs.5c16022)
Supplement: Supplementary file 2 [file ja5c16022_si_002.zip › All NMR FID Files/25/25_CarbonNMR/1/pdata/1/email_EM05-256-1-13C_1_1.pdf]

C13APT2048 CDCl3 /opt/nmrdata emeck 11

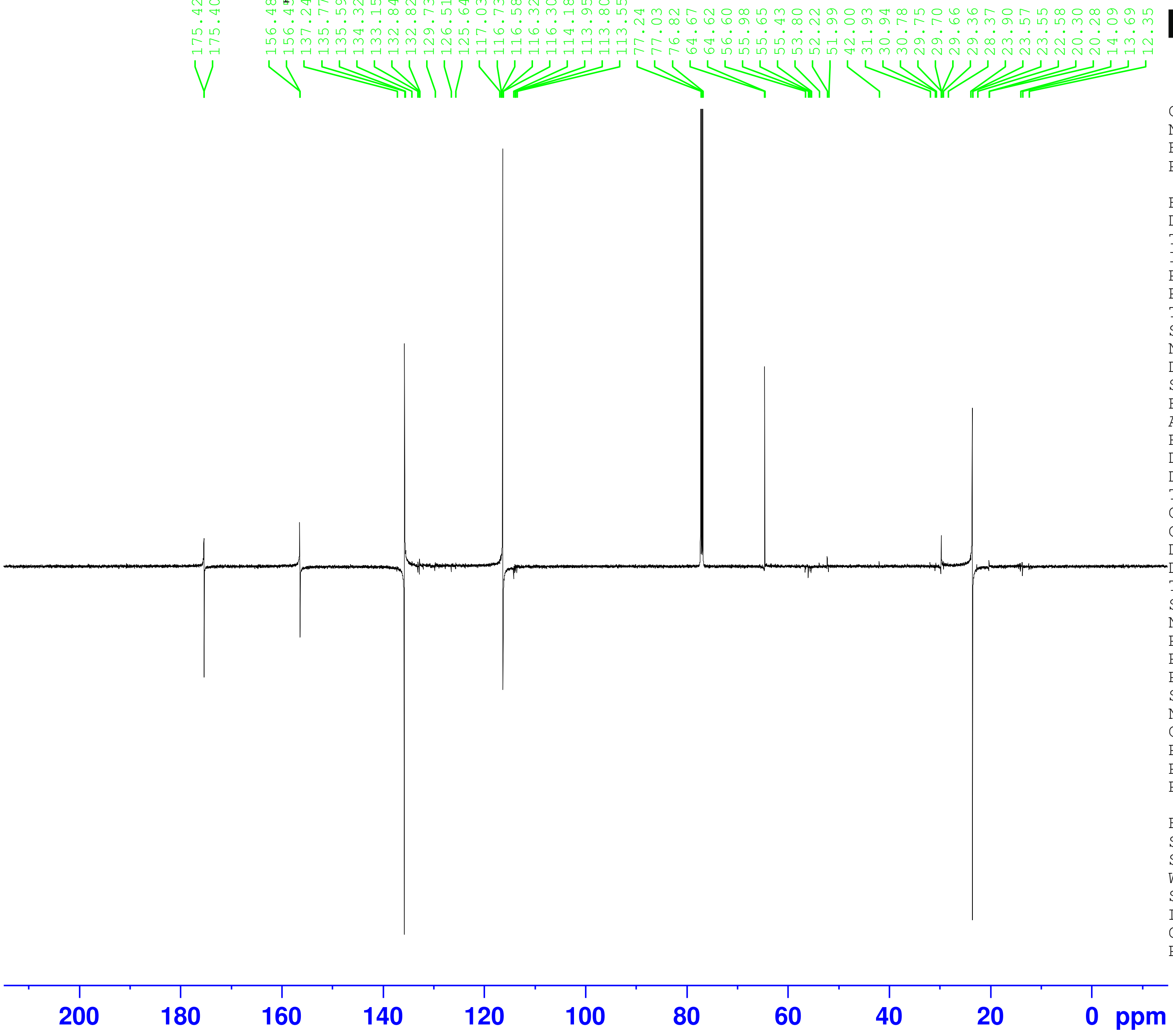

Current Data Parameters

NAME	EM05-256-1-13C
EXPNO	1
PROCNO	1

F2 - Acquisition Parameters

Date_	20240926
Time	22.45 h
INSTRUM	spect
PROBHD	Z168773_0053 (
PULPROG	jmod.acqt0
TD	65536
SOLVENT	CDCl3
NS	2048
DS	4
SWH	34722.223 Hz
FIDRES	1.059638 Hz
AQ	0.9437184 sec
RG	1030
DW	14.400 usec
DE	18.88 usec
TE	298.0 K
CNST2	145.0000000
CNST11	1.0000000
D1	5.00000000 sec
D20	0.00689655 sec
TD0	1
SFO1	150.9656791 MHz
NUC1	13C
P1	10.00 usec
P2	20.00 usec
PLW1	88.38300323 W
SFO2	600.3224013 MHz
NUC2	1H
CPDPRG[2	waltz16
PCPD2	80.00 usec
PLW2	21.85499954 W
PLW12	0.48474309 W

F2 - Processing parameters

SI	32768
SF	150.9505840 MHz
WDW	EM
SSB	0
LB	1.00 Hz
GB	0
PC	1.40
